# Drying kinetics, thermodynamic properties and physicochemical characteristics of Rue leaves

**DOI:** 10.1038/s41598-024-64418-5

**Published:** 2024-06-24

**Authors:** Geraldo Acácio Mabasso, Jennifer Cristhine Oliveira Cabral, Karine Feliciano Barbosa, Osvaldo Resende, Daniel Emanuel Cabral de Oliveira, Adrielle Borges de Almeida

**Affiliations:** 1 Federal Institute of Education, Science and Technology Goiano – Rio Verde Campus, Sul Goiana Street, Km 1, Zona Rural, Rio Verde, Goiás Brazil; 2 Faculty of Environmental Engineering and Natural Resources, Zambeze University, 7 de Abril Neighborhood, Regional Street 535, Km 5, Chimoio, Manica Mozambique

**Keywords:** *Ruta chalepensis* L., Mathematical modeling, Medicinal plants, Browning index, Antioxidant activity, Biochemistry, Plant sciences

## Abstract

Generally, medicinal plants are harvested with high amount of water, so it is essential to subject the product to drying as soon as possible to prevent degradation before application. Most compounds from medicinal plants are sensitive to drying processes, so it is important to adjust the drying conditions. The objective of this study was to describe the drying of Rue (*Ruta chalepensis* L.) leaves, select the models that best fit each drying condition, determine the activation energy and thermodynamic properties of the leaves, and evaluate their quality after drying. Leaves were harvested with moisture content of 3.55 ± 0.05 kg _water_ kg^−1^_dry matter_ and subjected to drying at temperatures of 40, 50, 60 and 70 °C. Valcam model showed the best fit to represent the drying kinetics of Rue leaves at temperatures of 40 and 70 °C, and Midilli model proved to be better for the temperatures of 50 and 60 °C. Effective diffusion coefficient increased linearly with the increase in drying air temperature, and the activation energy was 60.58 kJ mol^−1^. Enthalpy, entropy and Gibbs free energy values ranged from 57.973 to 57.723 kJ mol^−1^, from − 0.28538 to − 0.28614 kJ mol^−1^ K^−1^ and from 147.34 to 155.91 kJ mol^−1^, respectively, for the temperature range of 40–70 °C. Drying air temperature promoted darkening or tendency to loss of green color; increase in drying air temperature leads to greater discoloration, as well as a higher concentration of total phenolic compounds (about 221.10 mg GAE mL^−1^ g^–1^ dm), with a peak at temperature of 60 °C.

## Introduction

A large part of the world’s population has plants as an important source of treatment for various health problems, through their extracts. Medicinal and aromatic plants are widely used for the production of plant-based medicines, especially essential oils^1,2^. Kant & Kumar^1^ also state that, according to the WHO, there are 21 thousand species of plants with medicinal properties.

Rue (*Ruta chalepensis* L.) is a species of the genus *Ruta*, an aromatic medicinal plant popularly used as an herbal remedy for the treatment of various diseases. It belongs to the class of natural products with fungal, antioxidant, phytotoxic, abortifacient, antidote depressant and anti-inflammatory activity^3,4^. These properties (antioxidant, antimicrobial, and anti-inflammatory) constitute the main agents that confer the benefits of essential oils^5^. According to Conti et al.^6^, Rue essential oil extract can also be used satisfactorily as a larvicide and repellent for mosquitoes (*Aedes albopictus* Skuse), being very important in the fight against malaria, dengue and other viral diseases.

Plants of the *Rutaceae* family are native to the Mediterranean region and are distributed in several temperate and tropical countries^3,7,8^. These plants have stems of 30–70 cm in height and long lanceolate-type leaves^9^,besides a sympodial or cymose inflorescence, flowers composed of 4–5 sepals, 4–5 petals, 8–10 stamens and an upper ovary, with a strong deterrent odor. The species, popularly known as ‘arruda’ in Brazil, was introduced to the American continent after the arrival of the Spaniards, as part of the colonization process^10^.

According to Mejri et al.^11^, the essential oil is mainly concentrated in the shoots, particularly in fresh leaves, and drying is considered to be a reducing factor for most compounds, except for 2-tridecanone and camphor (absent in fresh leaves). Therapeutic properties of medicinal leaves are the main reason for studying the quality of dried leaves, including the contents of total phenolic compounds and antioxidants, as a function of the drying system adopted, especially in cases of use of high temperatures^5^.

In general, the moisture content in the leaves of medicinal plants is high, which leads to increments in metabolic activity and, consequently, physicochemical changes during storage. Therefore, after harvesting, it is important that leaves are subjected to drying to eliminate excess water, thus keeping metabolic activity low, allowing storage for a prolonged period, as well as breaking the seasonality of their availability^5,12^.

Removing excess water allows the product to be preserved for a long time. However, it is important to control the process so that quality is not compromised^2,13^, in addition to ensuring its greater efficiency. Téllez et al.^14^ argue that the drying process should be sufficiently fast, without compromising the quality of medicinal plants. Drying is affected by several factors, including drying air temperature, air velocity, and drying system, which can result in loss of bioactive compounds and color change^15^.

The drying process can be described by means of drying curves, depending on the drying conditions of each product. Mathematical modeling is an excellent tool for describing the drying process, with lots of application for estimating the drying time of the product and sizing equipment^16,17^. However, these curves are specific to each product and drying condition^18^, hence the need to study the behavior as a function of the product and associate it with quality parameters.

Several models have been proposed to satisfactorily describe the drying kinetics of various agricultural products^5,16,19,20,23,55^. Fitting helps to establish the best method and appropriate drying conditions, process description, drying time, and cost reduction, without significantly compromising physicochemical characteristics^12^.

Several authors have used different mathematical models to describe the drying process of agricultural products; however, as shown above, just a few focused on the description of drying process of Rue leaves, including the relation with physicochemical properties and drying condition, temperature and relative humidity.

In view of the above, the objective of this study was to describe the drying process of Rue leaves, select the models that best fit each drying condition, determine the activation energy and thermodynamic properties of the leaves, and evaluate their quality after drying based on the variation in color and chemical characteristics (lipid content, phenolics, flavonoids, and antioxidant activity). This issue is important to provide correct information about the drying process of Rue leaves without compromising the quality in terms of retaining the potentiality of their compounds, which could be compromised during the drying process.

## Material and methods

Rue (*Ruta chalepensis* L.) leaves were harvested in the experimental area belonging to Moporv—Planta & Vida (17° 48′ 04.96″ S; 50° 55′ 49.99″ W), located in the municipality of Rio Verde, Goiás, Brazil, with an initial moisture content of 3.55 ± 0.05 kg _water_ kg^−1^_dry matter_. The exsiccate of the plant is deposited under number HUFABC000885. Initial moisture content was determined by the oven method using three 15-g samples at a temperature of 103 ± 2 °C for 24 h, based on the difference between the initial and final mass.

After harvesting, the leaves were subjected to drying in a “Nova Ética” forced air circulation oven (model 400-3ND) until reaching the final moisture content for each condition and purpose at temperatures of 40, 50, 60 and 70 °C and relative humidity of 22.38, 10.07, 7.96 and 6.66%, respectively, commonly used to evaluate the effect of drying process of various agricultural products, such as leaves^36,51,54^. The relative humidity was monitored by a novus Logbox-RHT-LCD datalogger. The drying process occurred in two stages. In the first stage, the leaves were dried in four replicates, using metallic trays (120 mm × 49 mm in diameter and height) without perforation, containing 30.17 ± 0.11 g, until reaching the equilibrium moisture content for each drying condition. The airflow in the drying oven was in horizontal direction with homogeneous distribution to maintain the temperature. Drying was monitored by means of weighing at regular intervals, proportionally varied for each drying condition, on a semi-analytical balance (Shimadzu, model BL 3200H), with resolution of 0.01 g, until reaching constant mass, which corresponds to the following equilibrium moisture contents: 0.085 ± 0.003, 0.055 ± 0.003, 0.051 ± 0.001 and 0.037 ± 0.002 kg _water_ kg^−1^_dry matter_ for temperatures of 40, 50, 60 and 70 °C, respectively. In the second stage, aiming at the extraction of essential oil and determining its composition, drying was carried out until reaching the final moisture content of 0.16 ± 0.002 kg _water_ kg^−1^_dry matter_, which corresponds to the range considered safe for the long-term storage of medicinal plant leaves^21^. Drying data were then used to calculate the drying rate for each temperature, Eq. (1).

1 $${\text{DR}} = \frac{{\left( {{\text{X}}_{0} - {\text{X}}_{\rm i} } \right)}}{{\left( {{\text{t}}_{{{\rm i}}} - {\text{t}}_{0} } \right)}}$$

where DR: Drying rate (kg kg^−1^ h^−1^), X_0_: previous moisture content (decimal, d.b.), X_i_: current moisture content (decimal, d.b.), t_0_: total previous drying time (h), t_i_: total current drying time (h).

### Drying kinetics

Moisture content data obtained during drying for each condition, on a dry basis, were used to calculate the moisture content ratio, Eq. (2).

2 $$\text{RX} = \frac{\text{X} - {\text{X}}_{{\rm e}}}{{\text{X}}_{{\rm i}}-{\text{X}}_{{\rm e}}}$$

where RX: moisture content ratio (dimensionless), X: current moisture content (decimal, d.b.), X_e_: equilibrium moisture content (decimal, d.b.), X_i_: initial moisture content (decimal, d.b.).

The experimental values of RX were used to fit twelve mathematical models (Eqs. 3–14) commonly used to represent the drying kinetics of various agricultural products, considering each drying condition of Rue leaves^2,19,36^.

Approximation of diffusion^22^:

3 $${\text{RX }} = {\text{ a exp }}\left( { - {\text{k t}}} \right) \, + \, \left( {{1} - {\text{a}}} \right){\text{ exp }}\left( { - {\text{k b t}}} \right)$$

Henderson & Pabis^23^:

4 $${\text{RX }} = {\text{ a exp }}\left( { - {\text{k t}}} \right)$$

Logarithmic^24^:

5 $${\text{RX }} = {\text{ a exp }}\left( { - {\text{k t}}} \right)$$

Midilli^25^:

6 $${\text{RX }} = {\text{ a exp }}\left( { - {\text{k t}}^{{\text{n}}} } \right) \, + {\text{ b t}}$$

Newton^26^:

7 $${\text{RX }} = {\text{ exp }}\left( { - {\text{k t}}} \right)$$

Page^27^:

8 $${\text{RX }} = {\text{ exp }}\left( { - {\text{k t}}^{{\text{n}}} } \right)$$

Thompson^28^:

9 $${\text{RX }} = {\text{ exp }}\left\{ {\left[ { - {\text{a }} - \, \left( {{\text{a}}^{{2}} + {\text{ 4 b t}}} \right)^{{0.{5}}} } \right] \, / \, \left( {\text{2 b}} \right)} \right\}$$

Two terms^29^:

10 $${\text{RX }} = {\text{ a exp }}\left( { - {\text{k}}_{0} {\text{t}}} \right) \, + {\text{ b exp }}\left( { - {\text{k}}_{{1}} {\text{t}}} \right)$$

Two-term exponential^30^:

11 $${\text{RX }} = {\text{ a exp }}\left( { - {\text{k t}}} \right) \, + \, \left( {{1 } - {\text{ a}}} \right){\text{ exp }}\left( { - {\text{k a t}}} \right)$$

Valcam^31^:

12 $${\text{RX }} = {\text{ a }} + {\text{ b }} + {\text{ c t}}^{{{1}.{5}}} {\text{d t}}^{{2}}$$

Verna^32^:

13 $${\text{RX }} = - {\text{a exp }}( - {\text{k t}}) \, + \, ({1} - {\text{a}}){\text{ exp }}( - {\text{k}}_{{1}} {\text{t}})$$

Wang & Singh^33^:

14 $${\text{RX }} = { 1 } + {\text{ a t }} + {\text{ b t}}^{{2}}$$

where t: drying time, k, k_0_, k_1_: drying constants, a, b, c: model coefficients.

### Effective diffusion coefficient

Effective diffusion coefficient was determined considering each drying condition of Rue leaves, at temperatures of 40, 50, 60 and 70 °C, by fitting the mathematical model of the liquid diffusion for infinite flat slab, Eq. 15. This equation represents the analytic solution of Fick’s second law, considering the geometric shape of an infinite flat slab with eight-term approximation^34^.

15 $$\text{RX} = \frac{{\text{X}}-{\text{X}}_{{\rm e}}}{{\text{X}}_{{\rm i}}-{\text{X}}_{{\rm e}}}\text{=}\frac{8}{{\pi}^{2}}\sum_{{\rm n=1}}^{\infty}\frac{1}{{\left(\text{2n+1}\right)}^{2}}{\text{exp}}\left[\frac{{\left(\text{2n+1}\right)}^{2}{{\pi}}^{2}{{\text{D}}}_{{\rm ef}}\text{ t}}{4}{\left(\frac{\text{S}}{{\text{V}}}\right)}^{2}\right]$$

where t: drying time (s), D_ef_: liquid diffusion coefficient (m^2^ s^−1^), S: Equivalent leaf area (m), V: Equivalent leaf volume (m^3^), n: number of terms.

Area was obtained with a software program for determining the area of figures with irregular structure, by means of images, using 50 randomly selected leaves. Equivalent volume (Eq. 16) was determined by considering the equation of a triaxial spheroid by approximation, using the equation proposed by Mohsenin^35^. Thickness (L, m) was determined using a digital micrometer with resolution of 0.01 mm, measuring three points on each side of the midrib^36^.

16 $$\text{V} = \frac{{\pi} \, {\text{S}} \, {\text{L}}}{6}$$

Arrhenius equation (Eq. 17) was used to evaluate the influence of the drying air temperature by means of the effective diffusion coefficient. Applying the logarithm to the Arrhenius relation yields an expression that represents the linear function of Ln (D_ef_) as a function of the inverse of temperature (T^−1^) (Eq. 18), with angular coefficient that can be used to estimate the value of activation energy.

17 $${\text{D}}_{{\rm ef}}\text{=}{\text{D}}_{0}{\text{exp}}\left(\frac{-{\text{E}}_{{\rm a}}}{\text{RT}}\right)$$

18 $${\text{LnD}}_{{\rm ef}}\text{=}{\text{LnD}}_{0}-\left(\frac{{\text{E}}_{{\rm a}}}{\text{R}}\right)\frac{1}{{\text{T}}}$$

where D_0_: pre-exponential factor, R: universal gas constant (8.314 kJ kmol^−1^ K^−1^), T: temperature (K), E_a_: activation energy (kJ mol^−1^).

Fitting of the mathematical models of drying to the experimental data was performed through nonlinear regression analysis using the Gauss–Newton method. The degree of fit of each model was measured considering the magnitude of the adjusted coefficient of determination (R^2^), relative mean error (P, Eq. 19) and estimated mean error (SE, Eq. 20).

19 $$\text{P} = \frac{100}{{\text{n}}}\sum_{{\rm i=1}}^{\text{n}}\left(\frac{\left|\text{Y} - \widehat{\text{Y}}\right|}{\text{Y}}\right)$$

20 $$\text{SE} = \sqrt{\frac{\sum_{{\rm i=1}}^{\text{n}}{\left(\text{Y} - \widehat{\text{Y}}\right)}^{2}}{\text{DF}}}$$

where Y: value observed experimentally, Ŷ: value estimated by the model, n: number of experimental observations, DF: degree of freedom of the model (difference between the number of observations and the number of model parameters).

The selection of the models with the best fit was complemented using the Akaike Information Criterion (AIC) and the Shwarz’s Bayesian Information Criterion (BIC) (Eqs. 21 and 22), subjecting the pre-selected models to the Gauss–Newton criterion^16,19^.

21 $$\text{AIC} = -\text{2logL+2p}$$

22 $$\text{BIC} = -\text{2logL+pLn(n)}$$

where p and n: number of model parameters and observations, L: maximum likelihood, considering the estimates of the parameters.

### Thermodynamic properties

The thermodynamic properties related to the drying process of Rue leaves were determined by following the methodology described by Jideani & Mpotokwana^37^, widely applied to several agricultural products (Eqs. 23, 24 and 25)

23 $$\Delta{\text{H}}\text{=}{\text{E}}_{{\rm a}}-{\text{RT}}$$

24 $$\Delta\text{S=R}\left({\text{ln}}{\text{D}}_{0}-{\text{ln}}\frac{{\text{k}}_{{\rm B}}}{{\text{h}}_{{\rm p}}}-{\text{lnT}}\right)$$

25 $$\Delta\text{G} = \Delta\text{H}-\text{T}\Delta\text{S}$$

where ΔH: Enthalpy (J mol^−1^), ΔS: Entropy (J mol^−1^ K^−1^), k_B_: Boltzmann’s constant (1.38 × 10–^23^ J K^−1^), k_p_: Planck’s constant (6.626 × 10^–34^ J s^−1^), ΔG: Gibbs free energy (J mol^−1^).

### Color

The color of the Rue leaves was determined in a Color Flex EZ spectrophotometer, using three replicates for each drying air condition and fresh leaf. For the fresh leaves, color was evaluated on the adaxial and abaxial sides of the leaf blade. Readings of the L*, a* and b* coordinates obtained directly from the equipment were then converted into chromaticity (C*, Eq. 26), hue angle (H*, Eq. 27), color variation (ΔE, Eq. 28) and browning index (BI, Eq. 29)^2,38^. Hue angle of 0 or 360° indicates red hue, while angles of 270, 180 and 90° indicate blue, green and yellow hue, respectively^39^.

26 $${\text{C}}^{*}\text{=}\sqrt{{\left({\text{a}}^{*}\right)}^{2}\text{+}{\left({\text{b}}^{*}\right)}^{2}}$$

27 $${\text{H}}^{*}\text{=}\left\{\begin{array}{c}{\text{tan}}^{-{1}}\left({\text{b}}^{*}\text{/}{\text{a}}^{*}\right)\text{;} \, {\text{a}}\text{>}{0}\\ {180}\text{+} \, {\text{tan}}^{-{1}}\left({\text{b}}^{*}\text{/}{\text{a}}^{*}\right)\text{;} \, {\text{a}}\text{<}{0}\end{array}\right.$$

28 $$\Delta\text{E} = \sqrt{{\left({{\text{L}}^{*}}_{0}- \, {{\text{L}}^{*}}_{1}\right)}^{2}\text{+}{\left({{\text{a}}^{*}}_{0}- \, {{\text{a}}^{*}}_{1}\right)}^{2}\text{+}{\left({{\text{b}}^{*}}_{0}- \, {{\text{b}}^{*}}_{1}\right)}^{2}}$$

$$\text{BI} = \frac{\left[{100}\left({\text{x}}-\text{0.31}\right)\right]}{0.17}$$

29 $$\text{x} = \left({{\text{a}}^{*}}_{1}\text{+1.75}{{\text{L}}^{*}}_{1}\right)\text{/(5.645}{{\text{L}}^{*}}_{1}\text{+}{{\text{a}}^{*}}_{1}-\text{3.012}{{\text{b}}^{*}}_{1}\text{)}$$

where L^*^_0_, a^*^_0_, b^*^_0_: initial values (fresh leaf) for the coordinates L^*^, a^*^ and b^*^, L^*^_1_, a^*^_1_, b^*^_1_: values of the coordinates L^*^, a^*^ and b^*^ after drying, for each temperature, L^*^: corresponds to the variation from black to white, a^*^: chromatic coordinate with variation from green to red, b^*^: chromatic coordinate with variation from blue to yellow, C^*^: Chroma or saturation of the color; H^*^: hue angle (°); ΔE: color variation or change, BI: browning index.

### Antioxidant activity and total phenolic compounds

Antioxidant activity was determined by the removal of DPPH free radicals from Rue leaf extracts at different temperatures of the drying air and fresh leaf. The extract was prepared by adding 20 mL of 50% methanol to 1 g of crushed leaves for each condition. After 60 min of resting, the previously homogenized mixture was filtered and transferred to a 100 mL volumetric flask. The resulting residue was added with 20 mL of 70% acetone; after 60 min, it was filtered again to the same flask, and the volume was completed with distilled water.

Scavenging of DPPH (2,2-diphenyl-1-picrylhydrazyl) free radicals was determined by following the methodology of Brand-Williams et al.^40^, with some modifications. For this purpose, 3.9 mL of DPPH methanol solution (25 mg L^−1^) were added to a test tube containing 0.1 mL of the extracts and kept for 30 min in a dark environment at room temperature. After this period, the absorbance of the samples was determined in a UV/Visible spectrophotometer (UV-5100 Spectrophotometer, Metash) at wavelength of 515 nm (Abs_sample_) and a control absorbance of 80% methanol (Abs_control_), and the scavenging of free radicals was determined by the percentage of discoloration (Eq. 30).

Antioxidant activity was also determined using the ABTS method^41^, in triplicate. In a dark room, 30-µL aliquots of each dilution of the extract obtained previously were transferred into each test tube. Then, 3.0 mL of the ABTS radical [2,2′-azinobis-(3-ethylbenzothiazoline-6-sulfonic acid)], previously prepared and diluted in ethyl alcohol, were added to each test tube until an absorbance of 0.70 nm ± 0.05 nm was reached (control) using a UV/Visible spectrophotometer (UV-5100 Spectrophotometer, Metash) wavelength of 734 nm. The calibration of the spectrophotometer was carried out using ethyl alcohol. Then, the absorbance of the samples was determined after 6 min for each sample previously homogenized. And the result of free radical scavenging was also determined using the percentage of discoloration (Eq. 30).

30 $$\%\,Discoloration = \left[ {1 - \frac{{Abs_{{Sample}} }}{{Abs_{{control}} }}} \right] \times 100$$

Total phenolic compounds were determined using the same extract for antioxidant activity. For the quantification of total phenolics, 200 μL of the crude extract was added with 1.9 mL of Follin–Ciocalteau reagent diluted ten times in distilled water in a test tube. The same volume of aqueous solution of Na_2_CO_3_ (60 g L^−1^) was added to neutralize the mixture. After 120 min of reaction in the dark at room temperature, the absorbance of the samples was determined in a UV/Visible Spectrophotometer (Metash) at a wavelength of 725 nm and compared with a calibration curve of Gallic Acid Equivalent (GAE). The results were then expressed as mg GAE mL^–1^ g^−1^ of dry matter (dm)^42^.

### Statistical analysis

To evaluate the color of Rue leaves, a comparison was made between the fresh leaf and the leaf after drying for each temperature by Dunnett’s test at 5% significance level. Between the different temperatures, the data were subjected to linear regression analysis, at 5% significance level by the t-test. The data were also subjected to correlation analysis using Pearson’s correlation test, at 5% significance level.

## Results and discussion

### Modelling of drying process of Rue leaves

**Figure 1:**
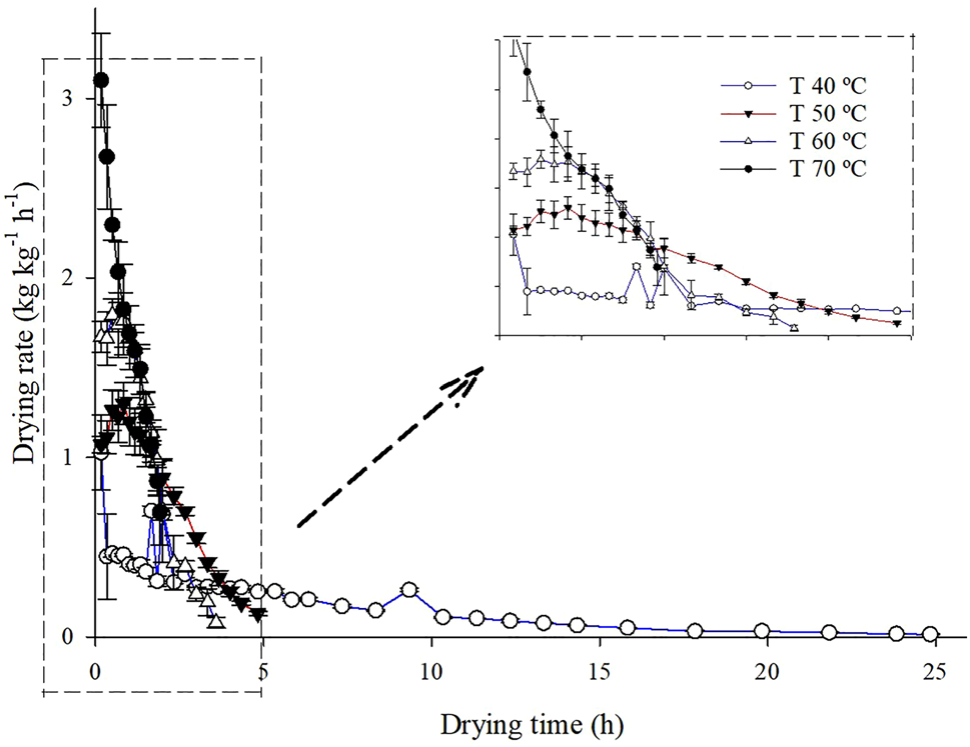
Drying rate during drying of Rue (*Ruta chalepensis* L.) leaves under different drying air conditions.

With the increase in drying air temperature, there was a reduction in the drying time of Rue leaves, from the initial moisture content of 3.55 ± 0.05 kg _water_ kg^−1^_dry matter_ to the final moisture content of 0.16 ± 0.002 kg _water_ kg^−1^_dry matter_. As the temperature increased, the drying rate showed a similar behavior to that of water loss, with reduction at low temperatures. The drying times to reach the final moisture content were 24.83, 4.83, 3.58, and 1.92 h for temperatures of 40, 50, 60, and 70 °C, respectively (Fig. 1). This behavior is consistent with the ease of water loss at high temperatures, especially in the initial phase of the process, when there is greater water availability.

In the final phase of the drying process, the values of the drying rate decreased, showing greater difficulty in removing water due to lower availability and lower speed for diffusion, making the process slow. According to Resende et al.^43^, the increase in drying time tends to favor a slow drying process as a consequence of the decrease in drying rate, since, with the increase in drying time, water is strongly bound to the dry matter, causing the minimum energy required for its evaporation to be greater. According to Lima et al.^44^, the lower the moisture content, the longer the time required for water to move from the center to the periphery by diffusion.

According to Babu et al.^5^, high gradient of moisture content and temperature leads to a higher drying rate, and the behavior depends on the material. The quality of dried leaves is strongly dependent on the drying time, so it is important that the drying process is fast enough to promote water loss and prevent the development of fungi. These aforementioned authors also state that the prolonged drying process has the potential to cause serious quality problems, thus altering the color, nutritional composition and structural organization.

Tables 1 and 2 show the values of the estimated mean error (SE), relative mean error (P), coefficient of determination (R^2^), Akaike Information Criterion (AIC) and Schwarz’s Bayesian Information Criterion (BIC) used to select the mathematical models with the best fit to describe the drying of Rue leaves at different drying air temperatures, by the Gauss–Newton criterion and AIC and BIC.

**Table 1:** Coefficient of determination (R^2^), relative mean error (P), estimated mean error (SE), Akaike Information Criterion (AIC) and Schwarz’s Bayesian Information Criterion (BIC) of Rue (*Ruta chalepensis* L.) leaves subjected to different drying air conditions.

Model	Temperature (°C)	SE	P (%)	R^2^	AIC	BIC
Approximation of diffusion	40	0.009	3.971	0.999	***	***
	50	0.023	12.678	0.998		
	60	0.009	4.244	0.999	***	***
	70	0.005	1.987	0.999	***	***
Henderson & Pabis	40	0.009	3.809	0.999	− 243.48	− 238.57
	50	0.039	23.853	0.993		
	60	0.040	21.197	0.986		
	70	0.045	35.707	0.991		
Logarithmic	40	0.009	3.922	0.999	− 246.49	− 239.94
	50	0.019	10.962	0.998		
	60	0.030	16.579	0.992		
	70	0.005	2.488	0.999	− 98.00	− 95.74
Midilli	40	0.009	3.973	0.999	− 243.58	− 235.40
	50	0.004	2.268	0.999	− 163.21	− 157.98
	60	0.008	2.426	0.999	− 115.71	− 111.26
	70	0.005	3.181	0.999	− 94.32	− 91.50
Newton	40	0.012	2.641	0.999	− 227.27	− 224.00
	50	0.048	30.708	0.989		
	60	0.048	27.977	0.978		
	70	0.046	39.085	0.989		

***Values not fitted by the AIC and BIC criteria.

**Table 2:** Coefficient of determination (R^2^), relative mean error (P), estimated mean error (SE), Akaike Information Criterion (AIC) and Schwarz’s Bayesian Information Criterion (BIC) of Rue (*Ruta chalepensis* L.) leaves subjected to different drying air conditions.

Model	Temperature (°C)	SE	P (%)	R^2^	AIC	BIC
Page	40	0.011	4.724	0.999	− 231.15	− 226.24
	50	0.007	3.601	0.999	− 145.62	− 142.49
	60	0.010	6.134	0.999	− 112.23	− 109.55
	70	0.027	19.108	0.997		
Thompson	40	0.012	3.502	0.999	− 233.33	− 228.41
	50	0.049	30.712	0.989		
	60	0.049	27.981	0.978		
	70	0.049	39.082	0.989		
Two terms	40	0.009	2.247	0.999	***	***
	50	0.286	64.990	0.565		
	60	0.042	21.196	0.986		
	70	0.284	67.157	0.629		
Two-term Exponential	40	0.012	3.308	0.999	− 225.48	− 220.57
	50	0.049	30.708	0.989		
	60	0.049	27.977	0.978		
	70	0.049	39.086	0.989		
Valcam	40	0.008	2.717	0.999	− 257.52	− 249.34
	50	0.008	4.830	0.999	− 140.29	− 135.07
	60	0.017	10.995	0.998		
	70	0.004	1.868	0.999	− 103.71	− 100.88
Verna	40	0.113	62.448	0.882		
	50	0.023	7.005	0.998	***	***
	60	0.033	18.625	0.991		
	70	0.027	20.470	0.997		
Wang & Singh	40	0.055	38.597	0.972		
	50	0.012	3.568	0.999	− 121.02	− 117.89
	60	0.019	9.277	0.997	− 88.20	− 85.53
	70	0.009	3.231	0.999	− 80.87	− 79.17

*** Values not fitted by the AIC and BIC criteria.

According to Mohapatra & Rao^45^, relative mean error values above 10% are not recommended for the selection of mathematical models of drying kinetics. From this perspective, considering the criterion of P, for each of the drying conditions, it was observed that all the models tested were fitted for at least one of the temperature conditions, and only the Midilli model (6) showed a good fit for all temperature conditions, followed by the Approximation of diffusion (3), Page (8), Valcam (12) and Wang & Singh (14) models, with favorable fit in at least three of the drying conditions.

According to Mohapatra & Rao^45^, models with P > 10% are excluded; however, this criterion cannot be adopted alone to define which model best describes the drying process. Criteria such as SE and R^2^ are also used together to define the best model. For these parameters, models with low SE and R^2^ values above 0.95 are considered to be better fitted. In this context, combining these elements, for all models with P < 10%, the R^2^ values were always greater than 0.95, so these models would be adequate to describe the drying of Rue leaves.

Regarding the SE value, for each drying condition, the following models stood out: Valcam (12) for temperatures of 40 and 70 °C, and Midilli for temperatures of 50 and 60 °C. As a way to strengthen the choice, other criteria are used in a complementary way, including AIC and BIC, and the model with the highest absolute values of AIC and BIC is considered to be best fitted. This criterion has been used satisfactorily, as it allows the selection of only one model among those pre-selected by the Gaussian-Newton method, since the parameters SE and R^2^ are not mutually exclusive.

According to AIC and BIC, the Valcam model (12) at temperatures of 40 and 70 °C and Midilli model at temperatures of 50 and 60 °C showed the highest absolute values of AIC and BIC, standing out among the others (Tables 1 and 2). For complementary analysis purposes, only those conditions pre-fitted by the Gaussian Newton method were considered. This criterion has satisfactorily stood out in the selection of models of drying kinetics of various agricultural products^16,19^.

**Figure 2:**
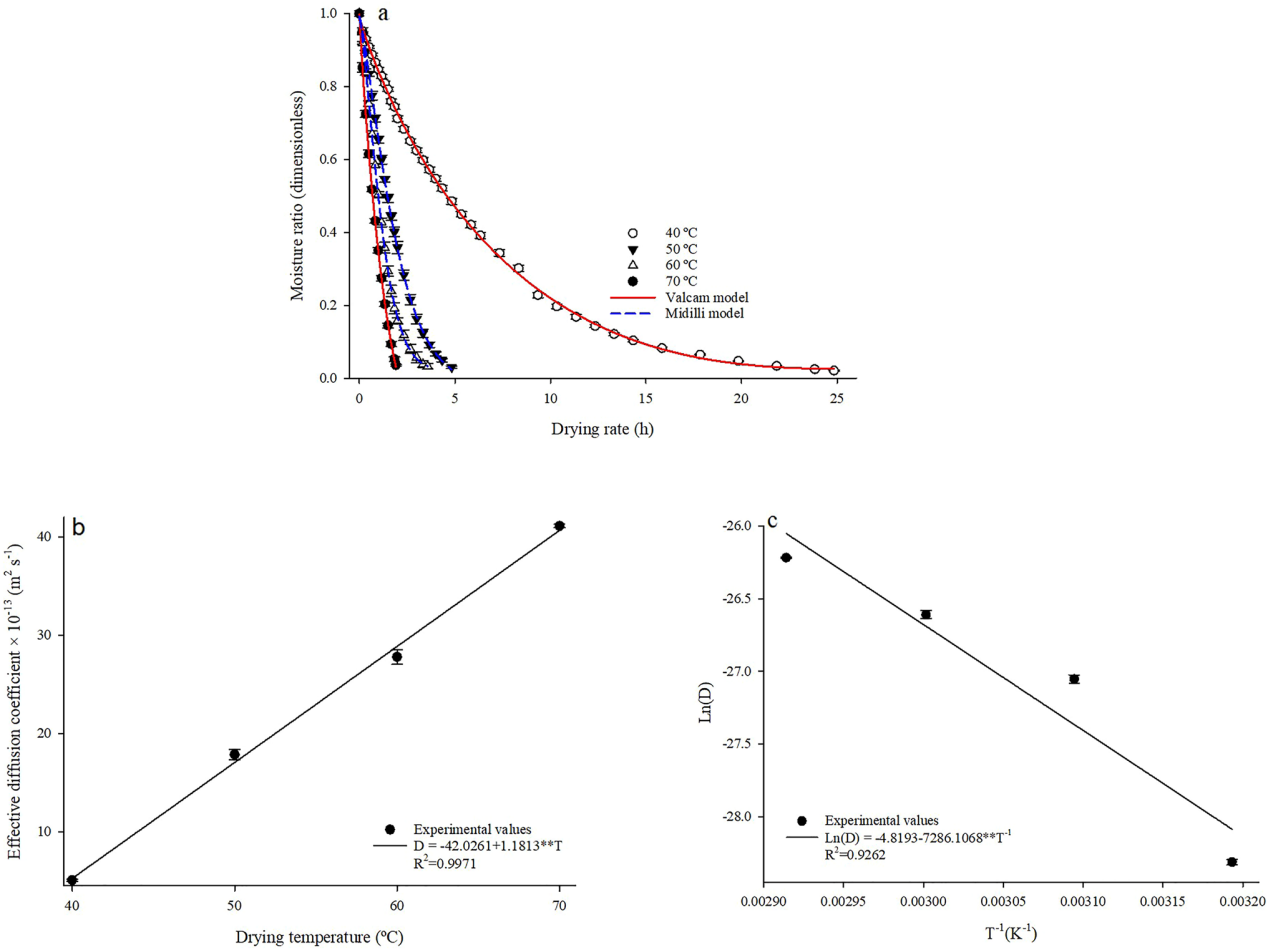
Values of the moisture content ratio estimated by the Valcam and Midilli models (**a**); effective diffusion coefficient (**b**); and Arrhenius representation for the effective diffusion coefficient (**c**) obtained for the drying of Rue (*Ruta chalepensis* L.) leaves at temperatures of 40, 50, 60 and 70 °C. ** Significant at p < 0.01 by t-test.

Figure 2a shows the mathematical modeling curves for the drying of Rue (*Ruta chalepensis* L.) leaves, represented by the models of Valcam (12) for temperatures of 40 and 70 °C and Midilli (06) for temperatures of 50 and 60 °C.

The selected models show a good fit to describe the drying process of Rue (*Ruta chalepensis* L.) leaves, due to the proximity between the observed and estimated values, as can be seen in the precision of the fit (Fig. 2a). The better the model’s trendline fits, the better the fit. Midilli model has been satisfactorily used to describe the drying kinetics of several leaves^5^ and other agricultural products under similar temperature conditions, especially *Momordica charantia* leaves^46^, *Stevia rebaudiana* leaves^47^, *Trigonella foenum-graecum* L. leaves^48^, *Morus nigra* L. leaves^13^, crushed mass of *Acmella oleracea* leaves^49^, *Artemisia absinthium* L. leaves^50^ and *Salvia officinalis* leaves^51^.

Table 3a shows the parameters of the Valcam model (12) at temperatures of 40 and 70 °C and Midilli model (06) for temperatures of 50 and 60 °C fitted to the drying of Rue (*Ruta chalepensis* L.) leaves.

**Table 3:** Coefficients for the Valcam (40 and 70 °C) and Midilli (50 and 60 °C) models (a); and enthalpy (ΔH), entropy (ΔS) and Gibbs free energy (ΔG) values for the different drying conditions of Rue (*Ruta chalepensis* L.) leaves (b).

Mathematical models (a)								
Temperature (°C)	Midilli				Valcam			
	a	b	k	n	a	b	c	d
40	–	–	–	–	0.991**	− 0.190**	0.045**	–0.003**
50	0.992**	− 0.004**	0.409**	1.301**	–	–	–	–
60	0.986**	0.005*	0.698**	1.394**	–	–	–	–
70	–	–	–	–	0.998**	− 1.018**	0.351**	0.015**

Thermodynamic properties (b)								
Temperature (°C)	ΔH (kJ mol^−1^)	ΔS (kJ mol^−1^ K^−1^)	ΔG (kJ mol^−1^)					
40	57.973	− 0.28538	147.341					
50	57.890	− 0.28564	150.196					
60	57.806	− 0.28590	153.053					
70	57.723	− 0.28614	155.913					

*Significant at p < 0.05 ** significant at p < 0.01 by t-test.

For the two selected models, it is observed that, in general, the values of the parameters show an increasing trend as a function of the drying air temperature, with emphasis on the parameter “k”, which represents the effective diffusivity of the drying in the falling rate period, and is therefore considered an approximation to explain the behavior of the drying air temperature^52^.

The value of “k” at both temperatures is consistent with the phenomenon of effective diffusivity of water during drying; however, in general, the behavior of the values of the other coefficients for the two models was random, being consistent with results obtained in other studies, for instance in the drying kinetics of *Morus nigra* L. leaves^13^ and crushed mass of *Acmella oleracea* leaves^49^.

**Figure 3:**
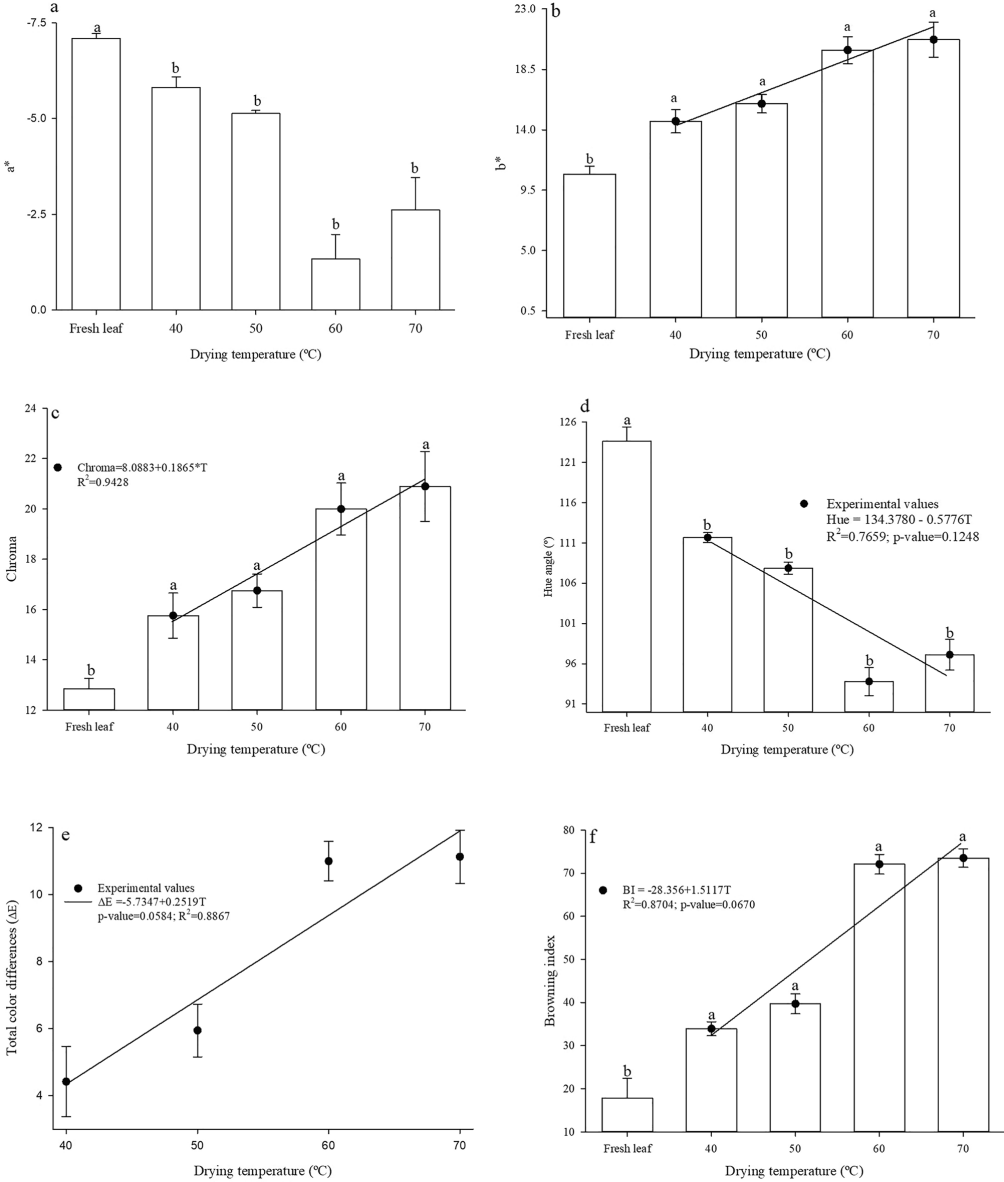
Mean values of a* (**a**), b* (**b**), chroma (**c**), hue angle (**d**), color variation (**e**) and browning index (**f**) of Rue (*Ruta chalepensis* L.) leaves after drying at different temperatures and fresh leaf. Significant at p < 0.01 by t-test. Pair of equal letters between the fresh leaf and each temperature condition do not differ from each other by Dunnett’s test at p < 0.05.

Figure 3b, c shows the values of the effective diffusion coefficient and the Arrhenius representation for Rue (*Ruta chalepensis* L.) leaves under different drying air temperature conditions. The experimental drying data were described by the equation based on Fick’s second law, considering the geometric shape of an infinite flat slab, with average thickness of 0.38 ± 0.78.

### Effective diffusion coefficient and thermodynamic properties of dried Rue leaves

The effective diffusion coefficient increased linearly with increasing drying air temperature, ranging from 5.0568 × 10^–13^ to 41.1173 × 10^–13^ m^2^ s^−1^ for temperatures of 40 to 70 °C, respectively (Fig. 2b). The reduction in temperature increases the internal resistance to water diffusion required to trigger the water evaporation process in the product. As a consequence, higher values of the effective diffusion coefficient were observed for higher temperatures.

The activation energy for the drying of Rue (*Ruta chalepensis* L.) leaves at temperatures of 40 to 70 °C was 60.5767 kJ mol^−1^. The value obtained is within the range established for biological products, which varies between 12.7 and 110 kJ mol^−1^^53^.

The effective diffusion coefficient is inversely proportional to the activation energy; the lower the activation energy, the higher the effective diffusion coefficient. Activation energy represents the minimum energy required to allow the movement of migration of water molecules during the drying process^54–56^.

The values of the activation energy in different biological materials vary, as they are dependent on the structure, chemical composition of the product, and the way the water is bound^53^. Silva et al.^54^ report that low activation energy values point to greater ease of water removal compared to other products subjected to the same drying conditions. The value obtained in this study is close to that obtained in the drying of *Morus nigra* L. leaves, which ranged from 65.94 to 66.08 kJ mol^−1^^13^ for the same temperature range and two drying air velocities.

In relation to the enthalpy values referring to the drying of Rue leaves, there was a reduction with the increase in drying air temperature, indicating a lower amount of energy needed to promote drying under conditions of high temperatures (Table 3b). This behavior is similar to that observed for entropy in absolute terms, for which there are also higher absolute values at high temperatures. The trend and negative values observed for entropy result in greater excitation of water molecules under conditions of elevated temperatures^57^ and chemical adsorption or structural changes of the adsorbent^58^.

Gibbs free energy showed an increasing trend with the increase in drying air temperature, suggesting that the drying process did not occur spontaneously, but rather due to the difference in partial pressure of the water vapor promoted by the heating of the air, thus forcing the water to exit the product, through the simultaneous process of gas exchange between the product and the surrounding air.

### Effect of different drying temperatures on the quality of dried Rue leaves

The values of the color parameter a* showed random behavior in relation to the different drying temperatures, although the trend of reduction is visible compared to the fresh leaf, because, for all drying conditions, the absolute values obtained at each temperature were always lower than those of the fresh leaf, showing a trend of change from green to red color, i.e., loss of green color (Fig. 3a).

Regarding the parameter b* (Fig. 3b), it was observed that the values obtained at different temperatures were always higher than those of the fresh leaf, showing a trend of change from blue to yellow color. The combination of the two parameters (a* and b*) clearly shows a tendency towards orange-red color. According to Doymaz & Karasu^51^, the variation in parameter b* is associated with the decomposition of chlorophyll and carotenoid pigments, non-enzymatic Maillard browning, and the formation of brown pigments, especially at elevated temperatures.

In relation to the parameter L*, which refers to lightness, the behavior was random, with no clear trend observed in relation to browning, despite a slight reduction mainly for temperatures of 60 and 70 °C, which would be consistent with the behavior observed in parameters a* and b*. A similar situation was also observed in a study on kinetics and color change of *Melissa officinalis* L. leaves^39^, in which the values fluctuated.

Chroma is related to saturation, and it was objectively verified that, with the increase in drying air temperature, chroma values also increased, as observed in comparison to the fresh leaf (Fig. 3c). This trend shows greater saturation in the color of the leaves, which can be associated with the reduction in the value of the L* parameter, making the leaves slightly dark at temperatures of 60 and 70 °C, contrasting with the opposite effect at lower temperatures.

The values of the hue angle decreased with the increase in drying air temperature and compared to the fresh leaf (Fig. 3d). The observed values show a tendency of variation or loss of green color of Rue leaves to orange-red color. Similar behavior was observed in leaves of *Melissa officinalis* L.^39^. On the other hand, Dermirhan & Ozbeck^38^ state that, when the values of the hue angle are greater than 90°, it is equivalent to saying that the leaves have not lost their green color, and values below 90° indicate that the leaves tend to have an orange-red hue. In this context, although a trend of reduction was observed, even at temperature of 70 °C, Rue leaves still retained a significant part of their green color, but in smaller proportions.

The variation increased with the increase in drying air temperature (Fig. 3e), showing that the tendency to darkening observed at the highest temperatures may have influenced the color between the fresh leaf and the dry leaves. This aspect is also highlighted by the behavior observed in the values of the browning index (Fig. 3f), which increased with increasing temperature, as well as in comparison to fresh leaves.

The color of medicinal and aromatic plants is a primary critical criterion for consumer preference, as it is associated with quality. Degradation or change in color, which includes browning, may be indicative of quality deterioration due to enzymatic reactions caused by polyphenol oxidase activity during the postharvest stages, especially drying^39^. According to Khallaf et al.^15^, temperature is considered to be the preponderant factor in the loss of bioactive compounds, mainly affecting color.

Rudy et al.^12^ also found changes in the color of *Dracocephalum moldavica* leaves, when subjected to different drying conditions and at different temperatures, as a consequence of the occurrence of enzymatic reactions and non-enzymatic browning, although no difference was found in relation to the L* coordinate for the different temperatures. However, the variation of color in comparison to the dry leaves and the temperatures adopted was clearly evident. On the other hand, the browning index reveals that there were indeed changes that corroborate the other parameters evaluated, as evidenced by the nature of the correlation between the browning index, with correlation coefficient values of 0.976, 0.976, 0.969, -0.968 and 0.981 for the parameters a*, b*, chroma, H* and ΔE, respectively.

Although the L* parameter was not correlated with the others, the negative sign in relation to ΔE and the browning index reveal that indeed the browning index and ΔE are inversely proportional to the magnitude of the L* parameter, thus reinforcing the lower values observed for temperatures of 60 and 70 °C.

**Figure 4:**
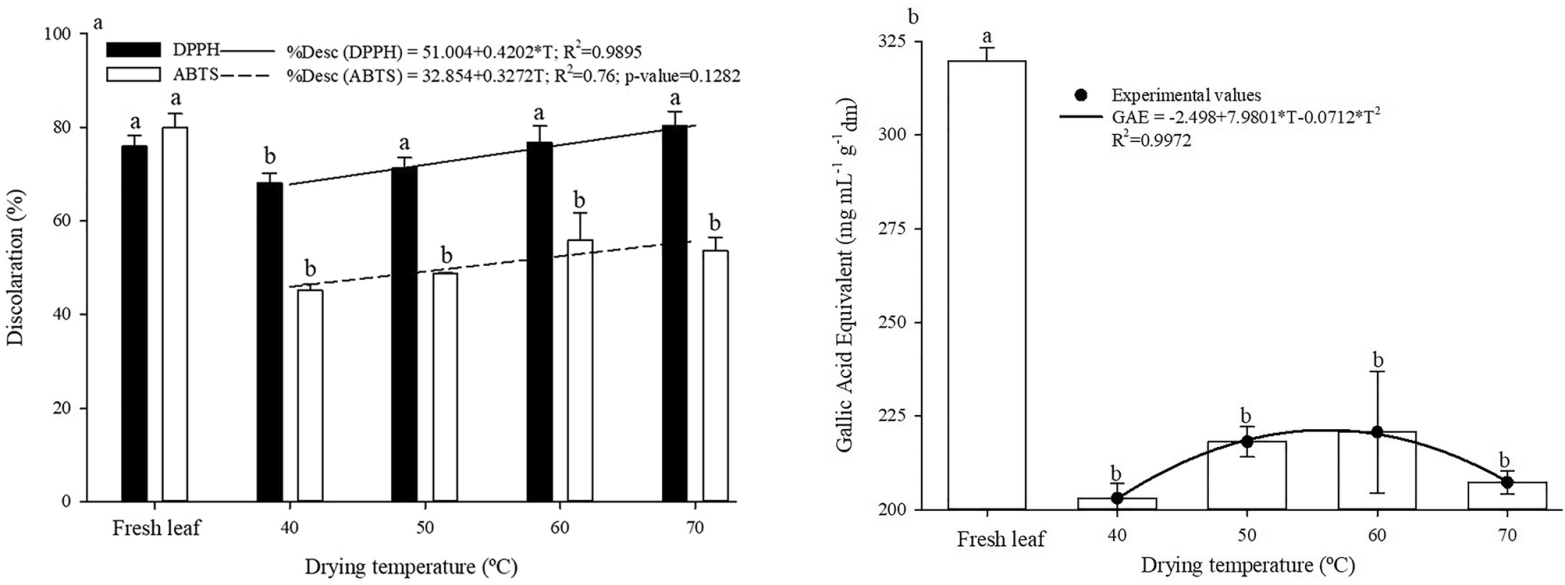
Mean values of the percentage of discoloration (**a**) and total phenolics (**b**) of Rue (*Ruta chalepensis* L.) leaves after drying at different temperatures and fresh leaf, on a dry basis. * Significant at p < 0.05 by t-test. Pair of equal letters between the fresh leaf and each temperature condition do not differ from each other by Dunnett’s test at p < 0.05.

Figure 4 shows the values referring to antioxidant activity, DPPH and ABTS free radical scavenging, by means of the percentage of discoloration (Fig. 4a) and total phenolics (Fig. 4b), through the GAE concentration on a dry basis. The discoloration values increased with the increase in drying air temperature, showing a positive effect of drying air temperature within the considered temperature range. This may be associated with the combined effect of temperature and drying time.

Regarding the concentration of total phenolic compounds, the values showed an increasing trend until reaching the temperature of 56.04 °C, which corresponds to the maximum concentration of total phenolic compounds, equivalent to 221.10 mg GAE mL^−1^ g^−1^ dm. In comparison to the fresh leaf, the temperature of 40 °C was less favorable to the antioxidant activity, while the concentration of total phenolics was lower for all temperatures studied.

Doymaz & Karasu^51^ state that the best drying conditions for medicinal plants should maximize the content of phenolic compounds. Thus, in a combined manner, aiming to maximize the concentration of phenolic compounds and antioxidant activity, the temperature close to 60 °C was favorable to the preservation of the quality of Rue leaves.

On the other hand, Babu et al.^5^ state that the temperature adopted for drying of leaves should be favorable to maintaining the quality of the dry product, promoting a more uniform drying process, in addition to ensuring its efficiency. The air temperature range of 40–60 °C is considered more suitable for drying leaves without significant loss of quality, which may justify the reduction of phenolic compounds from this temperature range close to 60 °C.

## Conclusions

Drying rate was higher for high drying air temperatures and decreased with drying time. Valcam model showed the best fit to represent the drying kinetics of Rue (*Ruta chalepensis* L.) leaves at temperatures of 40 and 70 °C, while Midilli model was the most suitable for temperatures of 50 and 60 °C. Effective diffusion coefficient increased linearly with the increase in drying air temperature, at the magnitude of 5.0568 × 10^–13^ to 41.1173 × 10^–13^ m^2^ s^−1^ for temperatures of 40 to 70 °C. The minimum energy required to trigger the drying process, represented by activation energy, was 60.5767 kJ mol^−1^ for Rue (*Ruta chalepensis* L.) leaves. Enthalpy, entropy and Gibbs free energy values ranged from 57.973 to 57.723 kJ mol^−1^, from − 0.28538 to − 0.28614 kJ mol^−1^ K^−1^ and from 147.341 to 155.913 kJ mol^−1^, respectively, for temperatures from 40 to 70 °C.

Drying air temperature influenced the color change, promoting darkening or tendency to loss of green color. Increase in drying air temperature led to greater discoloration, as well as a higher concentration of total phenolic compounds, with peak at temperature close to 60 °C. Drying is essential to prevent degradation of Rue leaves and other kinds of medicinal plants, providing condition for use for long times. For this purpose, it is important to conduct research that could confirm the potentiality reported for Rue leaves.

### Supplementary Information

Supplementary Information 1.

Supplementary Information 2.

## Data Availability

The data that support the findings of this study are included in this published article [and its supplementary information files]. Additional information is available from the corresponding author on reasonable request.
