# Supplementary material for: Drying kinetics, thermodynamic properties and physicochemical characteristics of Rue leaves
Source: Sci Rep. 2024 Jun 24;14:14526. doi: 10.1038/s41598-024-64418-5 (PMC11196716; doi:10.1038/s41598-024-64418-5)
Supplement: Supplementary file 1 — Supplementary Information 1. [file 41598_2024_64418_MOESM1_ESM.pdf]

SUPPLEMENTARY MATERIAL

Drying kinetics, thermodynamic properties and physicochemical characteristics of Rue leaves

Geraldo Acácio Mabasso[1,*,2], Jennifer Cristhine Oliveira Cabral[1], Karine Feliciano Barbosa[1], Osvaldo Resende[1], Daniel Emanuel Cabral de Oliveira[1], Adrielle Borges de Almeida[1]

* Corresponding author. email: geral.do@hotmail.com

[1] Federal Institute of Education, Science and Technology Goiano – Rio Verde Campus, Sul Goiana Street, km 1, Zona Rural, Rio Verde – Goiás, Brazil

[2] Faculty of Environmental Engineering and Natural Resources, Zambeze University, *7 de Abril* neighborhood, Regional Street 535, Km 5, Chimoio – Manica, Mozambique.

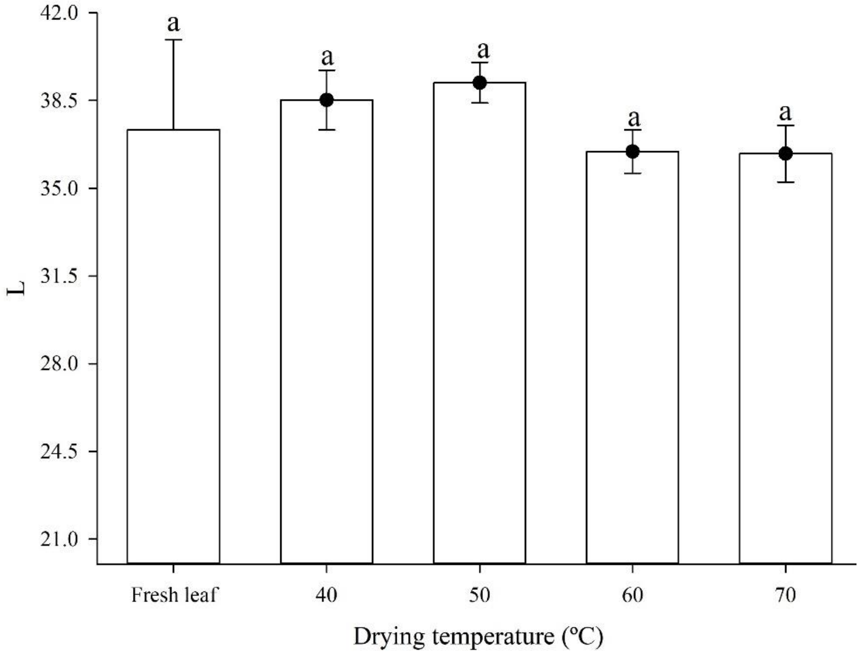

Pair of equal letters between the fresh leaf and each temperature condition do not differ from each other by Dunnett's test at $p<0.05$.

Figure a. Mean values of lightness (L) of rue (*Ruta chalepensis* L.) leaves after drying at different temperatures and fresh leaf

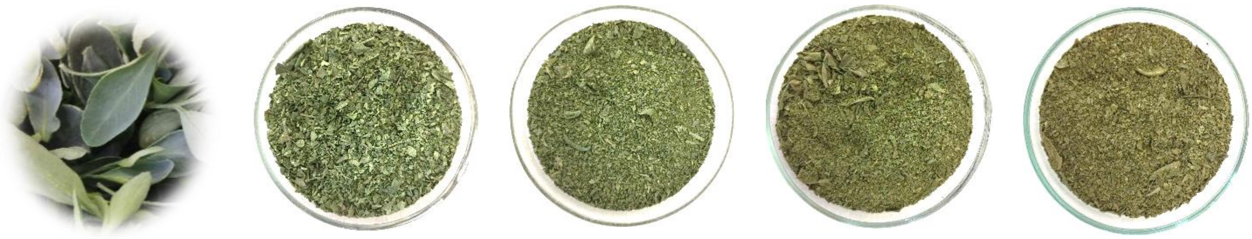

Fresh leaf T 40ºC T 50ºC T 60ºC T 70ºC

Figure b. *Ruta chalepensis* L. leaves before and after drying at different temperatures

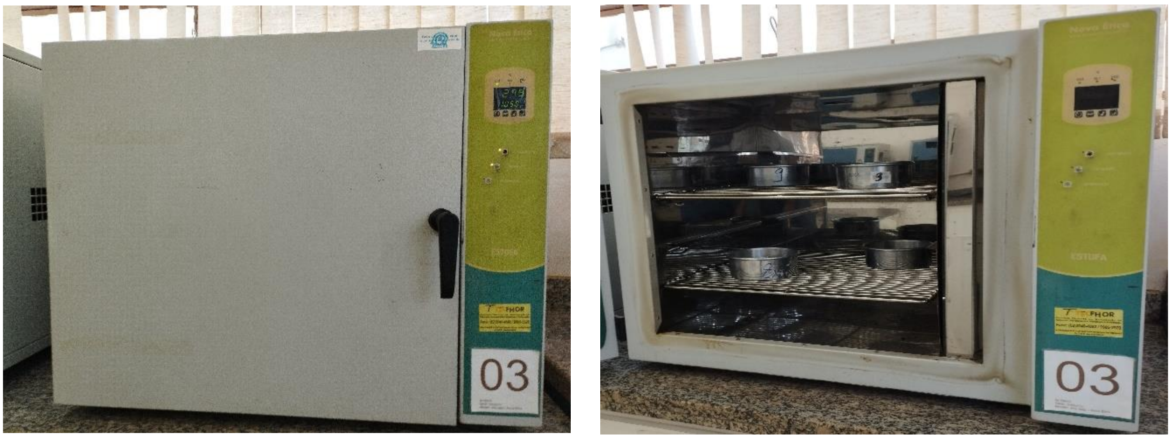

Figure c. Forced drying forced oven used to drying *Ruta chalepensis* L. leaves at different temperatures

Table a. Pearson's correlation coefficients between the color variables for rue (*Ruta chalepensis* L.) leaves subjected to different drying air temperatures

	a*	b*	L*	C	H*	ΔE	BI
a*		0.881**	-0.402^{ns}	0.856**	-0.967**	0.920**	0.976**
b*			-0.234^{ns}	0.997**	-0.952**	0.993**	0.976**
L*				-0.225^{ns}	0.309^{ns}	-0.229^{ns}	-0.388^{ns}
C					-0.929**	0.984**	0.969**
H*						-0.975**	-0.968**
ΔE							0.981**

*Significant at $p<0.01$ by t-test, ^{ns} not significant.
